# Propranolol restores susceptibility of XDR Gram-negative pathogens to meropenem and Meropenem combination has been evaluated with either tigecycline or amikacin

**DOI:** 10.1186/s12866-023-02934-6

**Published:** 2023-07-22

**Authors:** Samar S. Mabrouk, Ghada R. Abdellatif, Ahmed S. Abu Zaid, Khaled M. Aboshanab

**Affiliations:** 1 Department of Microbiology, Faculty of Pharmacy, Ahram Canadian University (ACU), 6Th October, Giza, Egypt; 2 Department of Microbiology & Immunology, Faculty of Pharmacy, Ain Shams University, Cairo, 11566 Egypt

**Keywords:** Extensive-drug-resistant, Carbapenem-resistant, Propranolol, Meropenem, Tigecycline, Or amikacin

## Abstract

**Background:**

Infection with extensive-drug-resistant (XDR) carbapenem-resistant (CR) Gram-negative bacteria (GNB) are viewed as a serious threat to human health because of the limited therapeutic options. This imposes the urgent need to find agents that could be used as adjuvants or combined with carbapenems to enhance or restore the susceptibility of XDR CR- GNB. Therefore, this study aimed to examine the effect of propranolol (PR) in combination with Meropenem (MEM) on the susceptibility profile of XDR CR-GNB recovered from severely infected patients as well as to evaluate combining MEM with either tigecycline (TGC) or amikacin (AK).

**Methods:**

A total of 59 non-duplicate CR- GNB were investigated for carbapenemase production by the major phenotypic methods. Molecular identification of five major carbapenemase-coding genes was carried out using polymerase chain reactions (PCR). Antimicrobial susceptibility tests were carried out using standard methods. Phenotypic and genotypic relatedness was carried out using the heatmap and ERIC PCR analysis. PR, 0.5 -1 mg/mL against the resulting non-clonal XDR CR-GNB pathogens were evaluated by calculating the MIC decrease factor (MDF). A combination of MEM with either AK or TGC was performed using the checkerboard assay.

**Results:**

A total of 21 (35.6%) and 38 (64.4%) CR-GNB isolates were identified as enterobacterial isolates (including 16 (27.1%) *Klebsiella Pneumoniae* and 5 (8.5%) *Escherichia coli)* and non-fermentative bacilli (including, 23 (39%), *Acinetobacter baumannii*, and 15 (25.4%) *Pseudomonas aeruginosa*)*.* The heatmap and ERIC PCR analysis resulted in non-clonal 28 XDR CR isolates. PR, at a concentration of 0.5 mg /ml, decreased MICs values of the tested XDR CR isolates (28; 100%) and restored susceptibility of only 4 (14.3%) isolates. However, PR (1 mg/mL) when combined with MEM has completely (28; 100%) restored the susceptibility of the tested XDR CR- GNB to MEM. The MEM + AK and MEM + TGC combination showed mostly additive effects (92.8% and 71.4%, respectively).

**Conclusion:**

PR at a concentration of 1 mg/mL restored the susceptibility of XDR CR- GNB to MEM which is considered a promising result that should be clinically investigated to reveal its suitability for clinical use in patients suffering from these life-threatening pathogens.

**Supplementary Information:**

The online version contains supplementary material available at 10.1186/s12866-023-02934-6.

## Background

Globally, the emergence and spread of extensive-drug resistant (XDR) carbapenem-resistant (CR) Gram-negative bacteria (GNB) are regarded as a grave threat to human health [1, 2]. The development of CR is currently gaining a great deal of attention since carbapenem antibiotics are considered the last line of defense in the face of serious XDR infections [1, 2]. The CR may result from reduced permeability of the outer membrane accompanied by overproduction of AmpC – beta-lactamases, development of extended-spectrum beta-lactamases [ESBLs), and excessive expression of class A, B, and D carbapenemase enzymes including *Klebsiella pneumoniae* carbapenemase (*bla*KPC), New Delhi metallo-β-lactamase (*bla*NDM), imipenemase (*bla*IMP), Verona integron encoded metallo-β- lactamase (*bla*VIM) and oxacillinases (*bla*OXA-48). This continues to be the most pathologically significant mechanism driving the evolution of resistance among the GNB globally and is responsible for the majority of nosocomial outbreaks in recent years [3, 4].

The high rates of morbidity and mortality, as well as the likelihood of widespread transmission of CR, particularly through transmissible genetic elements, make it imperative to quickly identify carbapenemase producers (CPs) in order to contain this serious public health crisis [5, 6]. Also, there is now a major concern on a global scale due to the explosive growth of XDR GNB, especially those that are CPs. Additionally, treatment options for pyogenic infections caused by XDR GNB pathogens have become extremely scarce and constrained [5, 6]. As a result, researchers have been concentrating on developing new treatments to deal with this health issue [7–9].

A number of recent reports proved propranolol (PR), a non-selective beta blocker, to have powerful negative effects on cell growth viability and progression and were evidenced to lessen cancer types [10, 11]. Recently, PR was analyzed to recover susceptibility of extensively drug-resistant (XDR) bacterial isolates to fluoroquinolones [12]. Moreover, combinations of MEM plus AK and MEM plus TGC have recently been widely used in clinical settings in Egypt after they have been tested as synergistic combinations[13, 14]. However, the pattern of XDR GNB particularly those which are CR are changeable by time which leads to an urgent demand to test the susceptibility of the circulating XDR pathogens [13, 14].

Although the respective antibiotic combinations have been widely used by clinicians, various septic infections caused by multidrug-resistant (MDR) or XDR CR- GNB have been documented recently causing high levels of morbidity and mortality worldwide [15–18]. Moreover, the effect of MEM in combination with PR on XDR CR- GNB isolates has not yet been explored. Accordingly, this study focused on investigating the effect of PR in combination with MEM on the susceptibility profile of XDR CR- GNB recovered from severely infected patients from two major Tertiary Care Hospitals in Egypt. This study also aimed to evaluate the efficacy of combining MEM with either TGC or AK on the respective life-threating pathogens.

## Materials and methods

### Identification and collection of clinical bacterial isolates

According to Bergey's manual of determinative bacteriology [19], the microscopic, morphologic, and biochemical characteristics were the basis of identification of the isolates. Additionally, the VITEK2 automated system (bioMérieux, Marcy L’Etoile, France) was used to further verify the bacterial identification [20]. All isolates were collected on typical workdays without the use of any specific exclusion criteria.

A total of 59 non-duplicate CR- GNB isolates (including 52 isolates were recovered from our previously conducted study in our lab [18], in addition to 7 isolates recently recovered in this study). All isolates were obtained from the discharged clinical specimens of unidentified patients from the microbiology laboratory of El Demerdash Tertiary Care Hospitals, Cairo, Egypt after the study approval by the Faculty of Pharmacy Ain Shams University Ethics Committee Number, ACUC-FP-ASU RHDIRB2020110301 REC# 41 in September 2021.

### The antimicrobial susceptibility testing of the collected bacterial isolates

The collected isolates were tested for antibiotic susceptibility using the Kirby-Bauer method, against thirteen different antibiotic discs (Thermo Scientific™ Oxoid™, Loughborough, UK); including amoxicillin/clavulanic acid (20 mg/10 mg), amikacin (30 mg), aztreonam (30 mg), cefoxitin (30 mg), ceftriaxone (30 mg), ciprofloxacin (5 mg), levofloxacin (5 mg), imipenem (10 mg), meropenem (10 mg), ertapenem (10 mg), sulphamethoxazole/trimethoprim (25 μg), doxycycline (30 μg) and tigecycline (30 μg). For each of the tested isolates, the Kirby-Bauer test was performed on Mueller–Hinton agar plates thrice, then, the inhibition zone diameters were measured. As previously reported, XDR isolates were determined using the international standard criteria of the CLSI guidelines, 2021 [21].

### Minimum inhibitory concentrations of the tested antibiotics

The XDR isolates demonstrating a pattern of resistance to any of the tested carbapenems were subsequently chosen to determine their MIC for MEM, AK, and TGC using the broth microdilution method according to CLSI guidelines, 2021 [22]. The broth microdilution test was performed in triplicates. According to CLSI, 2021, CR isolates with a high potential for carbapenemase production included Enterobacteriaceae isolates with MIC 4 g/mL and non-fermentative bacilli isolates with MIC 8 g/mL for MEM [22]. Reference strain of *E. coli* ATCC 25922™ was used for quality control monitoring.

### CPs phenotypic detection

#### Modified carbapenem inactivation method (mCIM)

The CLSI guidelines in 2021 recommended using mCIM for CPs detection using easily accessible laboratory reagents. Duplicate testing was done on XDR GNB isolates that were possibly CPs. A MEM disc was quickly submerged in a suspension prepared by suspending 1 µL loopful of the tested bacterial colonies in 2 mL TSB and then incubated for at least 4 h. The disc was then placed in an inoculated plate containing *E. coli* ATCC 25922™. The plate was incubated overnight and tested isolates with a zone of inhibition between 6 and 15 mm or colonies within 16–18 mm were classified as CPs. While isolates giving inhibition zones greater than or equal to 19 mm, were not classified as CPs [22].

#### Combined disk test

Combined disc test was carried out to identify the production of metallo-beta lactamases (MBLs) as previously reported [23]. The test was carried in duplicates to guarantee the reproducibility of results [23].

#### Blue-carba test

The Blue-carba test has a 100% sensitivity and specificity for direct detection of all CPs from bacterial culture. This test was carried out in the same manner as previously described by Pires et al. [24]. Duplicate testing was performed on potential CPs isolates to provide more reproducible results.

### Identification of carbapenemase-coding genes

Following the manufacturer's instructions, DNA of phenotypically confirmed XDR CP isolates were extracted using the Genomic DNA Purification Kit (Thermo Fisher Scientific, Waltham, MA, USA) and were used as templates for PCR using proper primers created by Macrogen® (Macrogen®, Madrid, Spain). The PCR amplification of the *bla*IMP, *bla*KPC, *bla*NDM, *bla*OXA-48, and *bla*VIM genes was performed using the annealing temperatures (Ta) and suitable primers as previously mentioned [18, 25]. The amplified PCR results were examined using agarose gel electrophoresis, and using a 1000 bp DNA ladder (GeneRuler 1 kb, ThermoFisher Scientific, Waltham, MA, USA).

### Phenotypic analysis using heatmap analysis

Antimicrobial resistance profiles, MIC to meropenem and carbapenemase production results were used to create a dendrogram showing heatmap signature of the isolates to illustrate their phenotypic relatedness. This was created by Morpheus online software (https://software.broadinstitute.org/morpheus/ accessed on 12 February 2023). using Euclidean distances as previously reported [26].

### Enterobacterial repetitive intergenic consensus-PCR (ERIC-PCR) for some selected clinical isolates

The ERIC-PCR sequence analysis tool is employed in epidemiological analysis to ascertain the genetic relatedness of bacterial isolates. The isolates' DNA template preparation was carried out in an accordance with Doyle et al. [27] and used as PCR templates. The primers (Table 1) and conditions utilized for the ERIC-PCR were explained by Codjoe et al..The following were the temperature and time conditions during ERIC-PCR: 35 cycles of primary denaturation at 94 °C for 5 min, secondary denaturation at 94 °C for 30 s, and annealing at 52 °C for 1 min, extension at 72 °C for 1 min, and final extension at 72 °C for 12 min [28]. The ERIC fingerprinting data was converted into a binary code based on the presence or absence of each band. Dendrograms were constructed using the unweighted pair group technique with arithmetic average (UPGMA) and Ward's hierarchical clustering routine using IBM® SPSS® version 23 of the Statistical Package for the Social Sciences software [29, 30]. Similarity index (Jaccard/Tanimoto Coefficient and number of intersecting elements) between all samples was computed using the online tool (https://planetcalc.com/1664/) (accessed on 20 January 2023). Agarose gel electrophoreses were carried out in accordance with Sambrook et al. with minor modifications [31].

**Table 1:** Resistance patterns to various antimicrobial agents among different tested carbapenem-resistant Gram-negative bacterial isolates (*n* = 59)

Antimicrobial class	Antimicrobial agent	Percentage of resistance (%)				
		***K. pneumoniae* (*n* = 16)**	***P. aeruginosa* (*n* = 15)**		***A. baumannii* (*n* = 23)**	***E. coli* (*n* = 5)**
Carbapenems	Imipenem (10 µg)	100	100	100		100
	Meropenem (10 µg)	100	100	100		100
	Ertapenem (10 µg)	100	ND	ND		100
β-lactam combination agents	Amoxicillin/clavulanic acid (20 μg/10 μg)	100	ND	ND		100
Monobactam	Aztreonam (30 µg)	100	100	ND		100
Cephalosporins	Cefoxitin (30 µg)	100	ND	ND		100
	Ceftriaxone (30 µg)	100	ND	100		100
Aminoglycosides	Amikacin (30 µg)	93.75	100	91.3		0
Fluoroquinolones	Ciprofloxacin (5 µg)	100	100	100		100
	Levofloxacin (5 µg)	100	100	100		100
Folate pathway inhibitors	Trimethoprim/sulfamethoxazole (25 µg)	93.75	ND	100		100
Tetracycline	Doxycycline (30 µg)	93.75	ND	95.7		60
Glycylcyclines	Tigecycline (30 µg)	93.75	ND	ND		40

*Abbreviations ND* not determined because they are not included in the reference guidelines (CLSI and EUCAST guidelines [32, 33]

### Evaluation of MEM- PR combinations

MEM combinations with PR, 25 μg-1 mg/mL (Sigma, Aldrich, UK), against the resulted non-clonal XDR CR- GNB pathogens were evaluated by determining the MIC decrease factor (MDF). The MDF of the tested isolates were estimated using the following equation:

MDF = MIC _without non-antibiotic_ / MIC _with non-antibiotic_.

An MDF value of 4 is regarded as a significant effect [34].

### Evaluation of MEM combinations with either AK or TGC

MEM was combined with additional antibiotics (AK and TGC) using the checkerboard assay to investigate the effect of such combinations on the in vitro MEM activity against the tested XDR GNB isolates [35, 36]. The fractional inhibitory concentration index (FIC index) was then estimated using the following equation and the results were interpreted as previously determined [35, 36].

$$\mathrm{FIC index}=\mathrm{FIC }\left(\mathrm{A}\right)+\mathrm{FIC }\left(\mathrm{B}\right)= \frac{\mathrm{MIC of A in combination}}{\mathrm{MIC of A alone}}+\frac{\mathrm{MIC of B in combination}}{\mathrm{MIC of B alone}}$$

### Statistical analysis

Statistical analysis, including descriptive statistics, frequency tables, cross-tabulations, dendrogram construction, and similarity index calculations were carried out using IBM® SPSS® version 23 of the Statistical Package for the Social Sciences software (SPSS Inc., Chicago, IL, USA). The Chi-square test was used to analyze categorical variables to assess statistical significance. A value of *P* < 0.05 was deemed statistically significant, and significance was two-sided.

## Results

### Identification of the recovered CR- GNB clinical isolates

Out of the collected 59 CR- GNB clinical isolates, 21 (35.6%) were recognized as enterobacterial isolates, including 16 (27.1%) *K. Pneumoniae* and 5 (8.5%) *E. coli*. Furthermore, a total of 38 (64.4%) were non-fermentative bacilli of which, 23 (39%) were *A. baumannii*, and 15 (25.4%) were *P. aeruginosa.*

### Antimicrobial susceptibility of the collected isolates

**Fig. 1:**
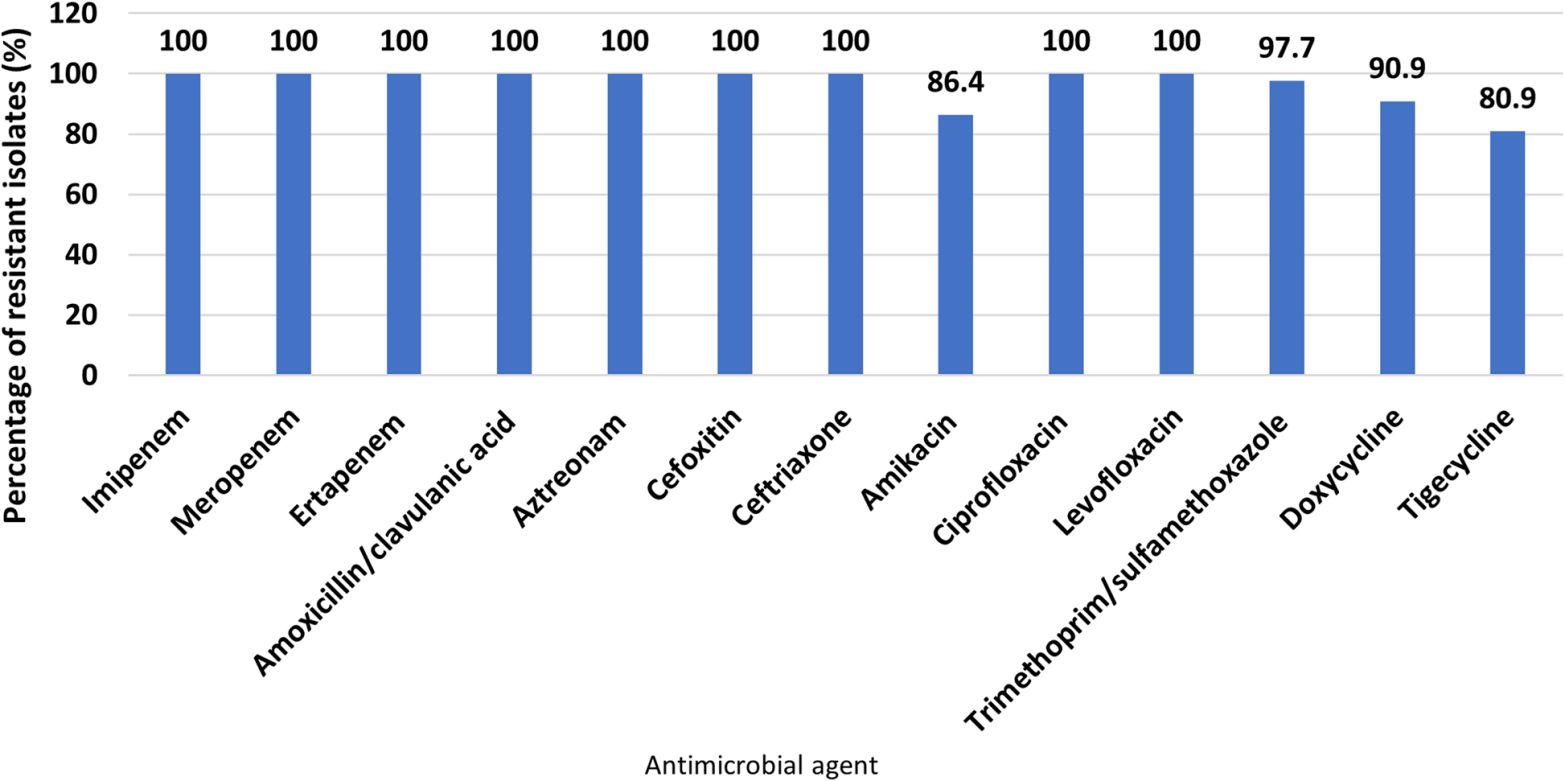
Prevalence of antimicrobial resistance of 59 carbapenem-resistant Gram-negative bacterial isolates to various tested antimicrobial agents. Prevalence was expressed as percent of resistant isolates relative to the total tested bacterial species for each antimicrobial agent

All of the 59 GNB isolates (100%) were proved to be resistant to one or more of the tested carbapenems and were categorized as CR-GNB isolates. The results of antimicrobial susceptibility testing showed that all the tested CR-GNB isolates were XDR, with the highest resistance rates to imipenem, meropenem, ertapenem, amoxicillin/clavulanic, aztreonam, cefoxitin, ceftriaxone, ciprofloxacin, and levofloxacin (100%). The lowest resistance rates were for TGC (80.9%), followed by AK (86.4%). The percentage of antimicrobial resistance of the 59 CR-GNB isolates are shown in Fig. 1, and their resistance patterns relative to identity are demonstrated in Table 1.

### Minimum inhibitory concentrations of the tested antibiotics

The MIC of MEM was determined against the 59 CR-GNB isolates. The MEM resistance was observed in all isolates, while the MICs of both the AK and TGC was tested against the selected non clonal 28 CR-GNB isolates according to the results obtained by the heatmap analysis. The AK resistance was observed in 23 out of 28 (82.1%) isolates, however, the 4 (14.3%) *P. aeruginosa* isolates tested in this assay were found to be resistant to TGC. Only 4 (14.3%) of the tested isolates were resistant to all three antibiotics in the assay; however, 19 (67.9%) isolates exhibited co-resistance to two antibiotics; 5 (17.9%) isolates exhibited MEM resistance only. The MICs of antimicrobial agents against the tested isolates are listed in Tables 2 and 3.

**Table 2:** The MICs of the tested antimicrobial agents, phenotypic and molecular analysis of Carbapenemase-encoding genes of the tested isolates (*n* = 28)

Isolate Code	Species	MIC of MEM (µg/ml)	Combined Disk Test	Modified Carbapenem Inactivation Test	Blue- Carba Test	Carbapenemase Genes
AB-9G	*A. baumannii*	256/R	-	-	+	*blaOXA-48*
AB-109A	*A. baumannii*	256/R	-	+	+	*blaOXA-48*
AB-63 M	*A. baumannii*	1024/R	-	+	+	*blaOXA-48, blaKPC*
AB-30G	*A. baumannii*	512/R	-	+	+	*blaKPC, blaVIM*
AB-100 M	*A. baumannii*	1024/R	-	-	+	*blaKPC*
AB-55 M	*A. baumannii*	1024/R	-	+	+	*blaOXA-48*
AB-20G	*A. baumannii*	256/R	+	+	+	*blaVIM*
AB-3 M	*A. baumannii*	512/R	+	+	+	*blaOXA-48, blaKPC*
AB-14 M	*A. baumannii*	128/R	-	+	+	*blaOXA-48, blaKPC, blaVIM*
AB-37 M	*A. baumannii*	1024/R	+	+	+	*blaVIM*
AB-34 M	*A. baumannii*	128/R	-	+	+	*blaKPC*
AB-13 M	*A. baumannii*	512/R	+	+	-	*blaVIM*
AB-7G	*A. baumannii*	512/R	+	-	+	*blaKPC*
AB-6 M	*A. baumannii*	512/R	-	+	+	*blaOXA-48, blaKPC*
AB-36 M	*A. baumannii*	128/R	+	+	+	*blaVIM*
AB-28 M	*A. baumannii*	512/R	+	+	+	*blaOXA-48, blaKPC*
AB-42 M	*A. baumannii*	64/R	+	+	+	*blaOXA-48, blaKPC*
AB-74 M	*A. baumannii*	128/R	+	+	+	*blaOXA-48, blaKPC*
AB-13G	*A. baumannii*	1024/R	+	-	+	*blaOXA-48, blaKPC, blaVIM*
AB-64 M	*A. baumannii*	128/R	+	+	+	*blaOXA-48, blaKPC*
AB-30 M	*A. baumannii*	128/R	+	+	+	*blaOXA-48, blaKPC*
AB-15 M	*A. baumannii*	64/R	+	+	+	*blaOXA-48, blaKPC*
AB-31G	*A. baumannii*	128/R	-	+	+	*blaKPC, blaVIM*
KP-84S	*K. pneumoniae*	64/R	-	+	+	*blaOXA-48, blaKPC*
KP-25S	*K. pneumoniae*	1024/R	-	+	+	*blaKPC*
KP-89S	*K. pneumoniae*	128/R	-	+	+	*blaOXA-48, blaKPC*
KP-92A	*K. pneumoniae*	1024/R	+	+	+	*blaOXA-48, blaKPC, blaVIM*
KP-114S	*K. pneumoniae*	1024/R	+	+	+	*blaOXA-48, blaKPC*
KP-106S	*K. pneumoniae*	512/R	-	+	+	*blaKPC*
KP-79A	*K. pneumoniae*	512/R	+	+	+	*blaKPC*
KP-113S	*K. pneumoniae*	512/R	-	+	+	*blaOXA-48, blaKPC*
KP-81S	*K. pneumoniae*	256/R	-	+	+	*blaVIM*
KP-11 K	*K. pneumoniae*	128/R	+	+	+	*blaVIM*
KP-7 K	*K. pneumoniae*	512/R	+	+	+	*blaOXA-48, blaKPC*
KP-4 K	*K. pneumoniae*	512/R	+	+	+	*blaVIM*
KP-9*	*K. pneumoniae*	1024/R	+	+	+	*blaKPC*
KP-112S	*K. pneumoniae*	1024/R	+	-	+	*blaVIM*
KP-78A	*K. pneumoniae*	64/R	+	+	+	*blaKPC*
KP-93A	*K. pneumoniae*	128/R	+	+	+	*blaOXA-48, blaKPC, blaVIM*
PA-33 K	*P. aeruginosa*	1024/R	-	+	+	*blaKPC*
PA-59S	*P. aeruginosa*	128/R	-	+	+	*blaOXA-48*
PA-51S	*P. aeruginosa*	1024/R	-	+	+	*blaOXA-48, blaKPC*
PA-19S	*P. aeruginosa*	64/R	-	+	+	*blaOXA-48*
PA-100S	*P. aeruginosa*	1024/R	-	+	+	*blaOXA-48*
PA-78S	*P. aeruginosa*	128/R	-	+	+	*blaOXA-48*
PA-54S	*P. aeruginosa*	1024/R	-	+	-	*blaKPC*
PA-99S	*P. aeruginosa*	1024/R	-	-	+	*blaKPC*
PA-50S	*P. aeruginosa*	256/R	+	+	+	*blaOXA-48*
PA-83S	*P. aeruginosa*	128/R	-	+	+	*blaOXA-48, blaKPC*
PA-18S	*P. aeruginosa*	128/R	+	+	+	*blaVIM*
PA-77Y	*P. aeruginosa*	64/R	+	+	+	*blaOXA-48, blaVIM*
PA-111S	*P. aeruginosa*	1024/R	+	+	+	*blaKPC*
PA-98S	*P. aeruginosa*	64/R	-	-	+	*blaKPC*
PA-79S	*P. aeruginosa*	1024/R	-	+	+	*blaOXA-48*
EC-53A	*E. coli*	512/R	-	+	+	*blaKPC*
EC-55A	*E. coli*	512/R	-	+	+	*blaKPC*
EC-99A	*E. coli*	256/R	-	+	+	*blaKPC*
EC-34R	*E. coli*	64/R	-	+	+	*blaKPC*
EC-98A	*E. coli*	64/R	-	+	+	*blaKPC*

*Abbreviations MIC* Minimum inhibitory concentration, *MEM* Meropenem, *AK* amikacin, *TGC* Tigecycline; *R* Resistant, *I* Intermediate sensitivity, *S* Susceptible, *AB A. baumannii, KP K. pneumoniae, PA P. aeruginosa, EC E. coli*

**Table 3:** Effects of PR on the MIC of Meropenem

Isolate code	MICs (μg/ml) MEM alone	PR 0.5 mg /ml		PR 1 mg /ml	
		**MICs (μg/ml) MEM + PR 0.5 mg /ml**	**MDF**	**MICs (μg/ml) MEM + PR 1 mg /ml**	**MDF**
AB-9G	256	32	8	2	128
AB-109A	256	16	16	1	256
AB-63 M	1024	32	32	2	512
AB-30G	512	256	2	1	512
AB-100 M	1024	32	32	2	512
AB-55 M	1024	512	2	1	1024
AB-20G	256	8	32	2	128
AB-3 M	512	8	64	1	512
AB-37 M	1024	32	32	2	512
AB-34 M	128	64	2	0.5	256
AB-14 M	128	8	16	2	64
AB-13 M	512	256	2	2	256
AB-7G	512	8	64	0.5	1024
KP-84S	64	2	8	2	128
KP-89S	128	2	64	0.5	256
KP-114S	1024	64	16	1	1024
KP-25S	1024	128	8	4	256
KP-92A	1024	32	32	4	256
KP-106S	512	32	16	1	512
KP-79A	512	32	16	1	512
KP-113S	512	64	8	2	256
PA-50S	256	4	64	1	256
PA-99S	1024	64	16	4	256
PA-54S	1024	32	32	2	512
PA-78S	128	4	32	0.5	256
EC-55A	512	64	8	4	128
EC-34R	64	8	8	0.5	128
EC-99A	256	16	16	1	256

*Abbreviations*: *MICs* minimum inhibitory concentrations, *MDF* MIC decrease factor, *MEM* Meropenem, *PR* Propranolol, *AB A. baumannii, KP K. pneumoniae, PA P. aeruginosa, EC E. coli*

### Phenotypic detection of XDR CR- GNB

As shown in Table 2, the carbapenemase producing XDR GNB (*n* = 59) isolates were phenotypically assessed using the combined disk test, modified carbapenem inactivation method (m CIM), and blue-carba test.

### Molecular detection of Carbapenemase Producers (CPs)

Multiplex PCR results revealed that *bla*KPC was amplified in 40 (67.8%) isolates (16 *A. baumannii*, 12 K*. pneumoniae*, 7 *P. aeruginosa* and 5 *E. coli*), followed by *bla*OXA-48 that was detected in 30 (50.8%) isolates (14 *A. baumannii*, 7 K*. pneumoniae* and 9 *P. aeruginosa*), followed by *bla*VIM that was observed in 16 (27.1%) isolates (8 *A. baumannii* and 6 K*. pneumoniae* and 2 *P. aeruginosa*). However, none of the tested XDR isolates had any *bla*IMP or *bla*NDM. Data summary of MICs, phenotypic and genotypic findings of the XDR GNB (*n* = 59) is tabulated in Table 2.

### Heatmap analysis of XDR GNB isolates

**Fig. 2:**
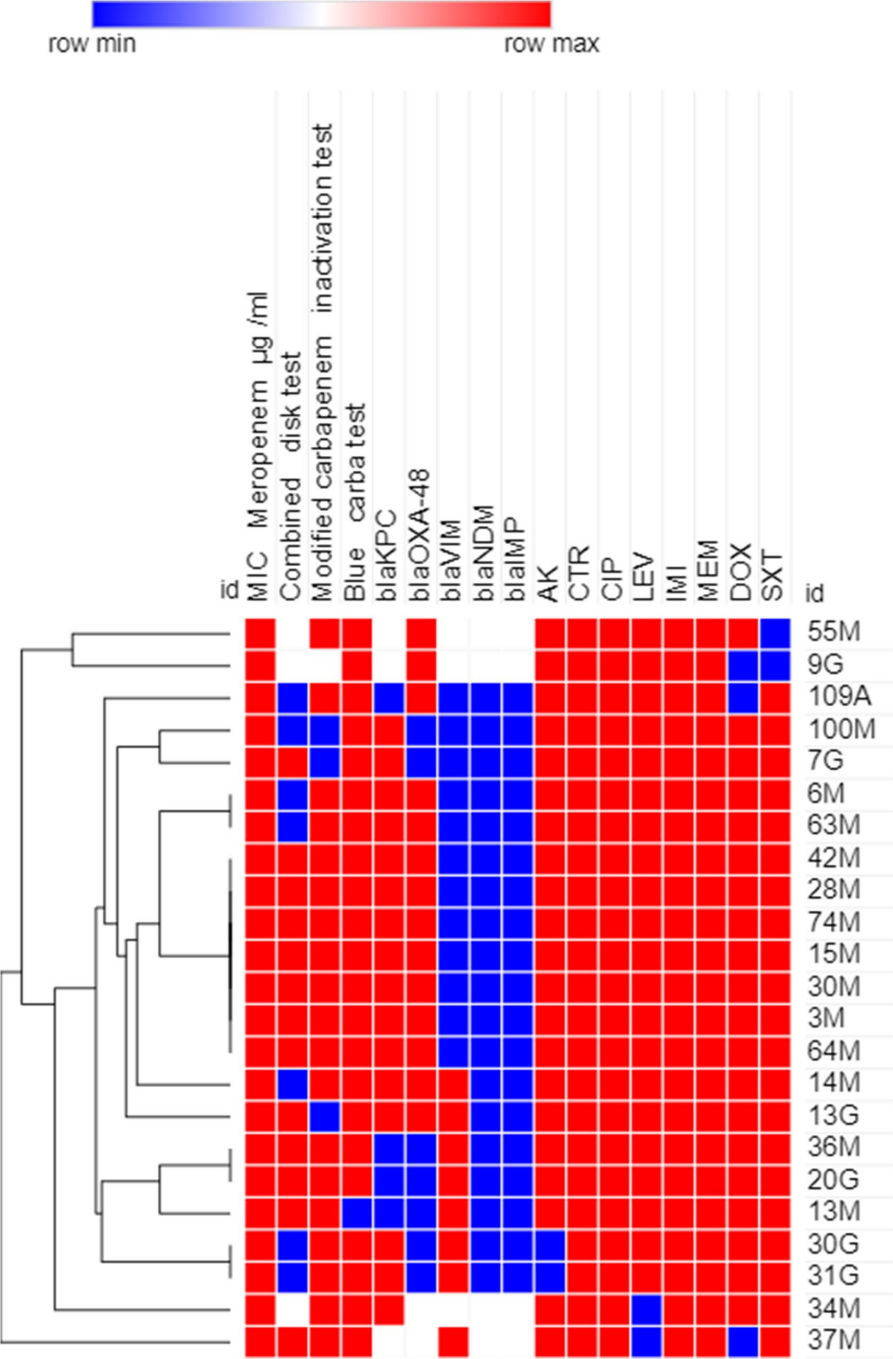
Heatmap of carbapenem-resistant *A. baumnnii* isolates (*n=23*) in this study based on their antimicrobial resistance patterns, MIC to meropenem and phenotypic tests for carbapenemase enzyme production (Combined disk test, Modified carbapenem inactivation method and Blue-Carba test results). This heatmap was generated by using Morpheus online software using Euclidean distances (https://software.broadinstitute.org/morpheus/). Blue color indicates Positive or resistant; Red color indicates negative or sensitive, White color indicates Intermediate resistance. AK, Amikacin; CTR, Ceftriaxone; CIP, Ciprofloxacin; LEV, Levofloxacin; IMI, Imipenem; MEM, Meropenem; DOX, Doxycycline; SXT, Trimethoprim-Sulfamethoxazole

**Fig. 3:**
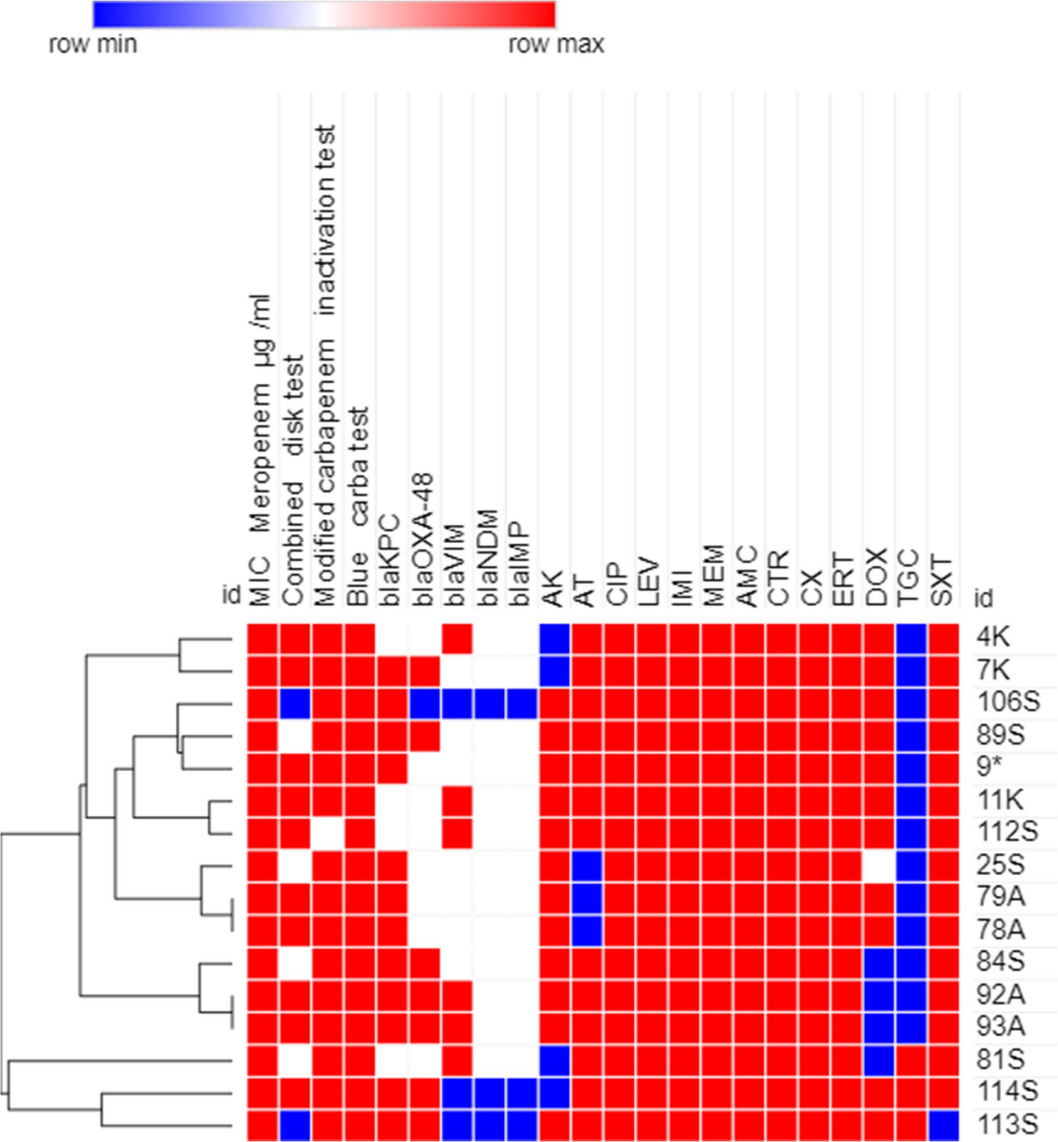
Heatmap of carbapenem-resistant *K. pneumoniae* isolates (*n=16*) in this study based on their antimicrobial resistance patterns, MIC to meropenem and phenotypic tests for carbapenemase enzyme production (Combined disk test, Modified carbapenem inactivation method and Blue-Carba test results). This heatmap was generated by using Morpheus online software using Euclidean distances (https://software.broadinstitute.org/morpheus/). Blue color indicates Positive or resistant; Red color indicates negative or sensitive, White color indicates Intermediate resistance. AK, Amikacin; AT, Aztreonam; CIP, Ciprofloxacin; LEV, Levofloxacin; IMI, Imipenem; MEM, Meropenem; AMC, Amoxicillin-clavulanic acid; CTR, Ceftriaxone; CX, Cefoxitin; ERT, Ertapenem; DOX, Doxycycline; TGC, Tigecycline; SXT, Trimethoprim-Sulfamethoxazole

**Fig. 4:**
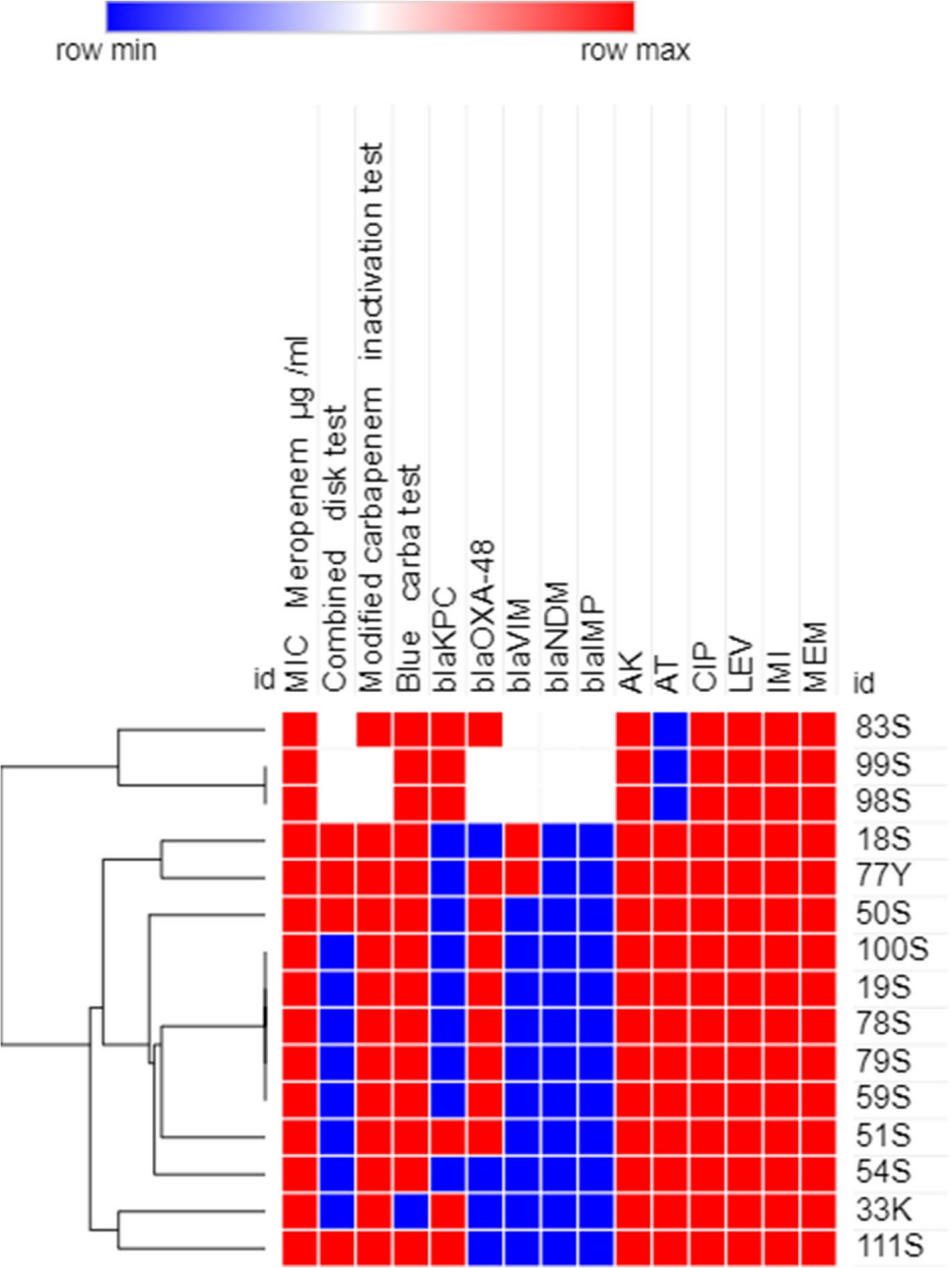
Heatmap of carbapenem-resistant *P. aeruginosa* isolates (*n=15*) in this study based on their antimicrobial resistance patterns, MIC to meropenem and phenotypic tests for carbapenemase enzyme production (Combined disk test, Modified carbapenem inactivation method and Blue-Carba test results). This heatmap was generated by using Morpheus online software using Euclidean distances (https://software.broadinstitute.org/morpheus/). Blue color indicates Positive or resistant; Red color indicates negative or sensitive, White color indicates Intermediate resistance. AK, Amikacin; AT, Aztreonam; CIP, Ciprofloxacin; LEV, Levofloxacin; IMI, Imipenem; MEM, Meropenem

**Fig. 5:**
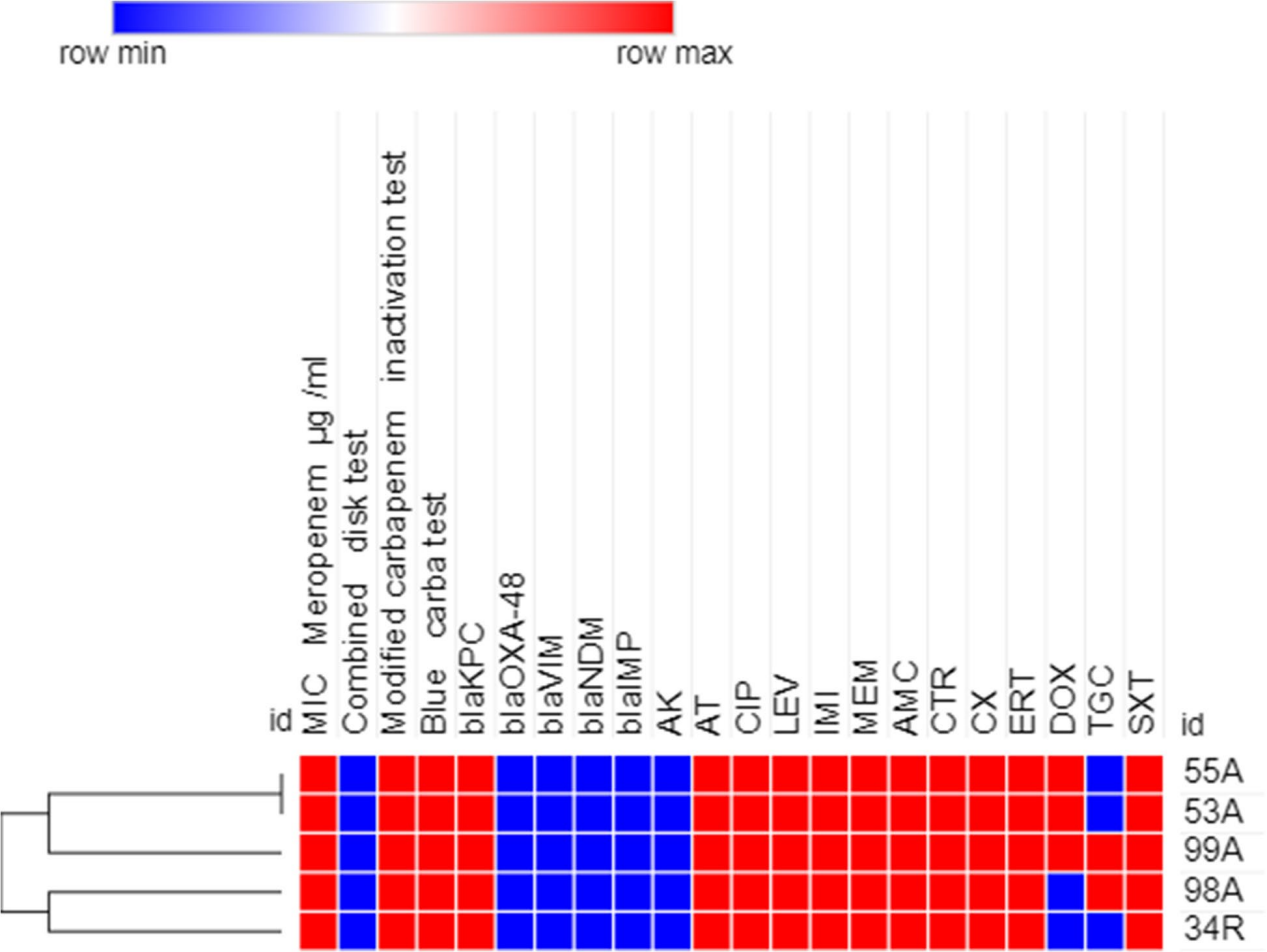
Heatmap of carbapenem-resistant *E. coli* isolates (*n=5*) in this study based on their antimicrobial resistance patterns, MIC to meropenem and phenotypic tests for carbapenemase enzyme production (Combined disk test, Modified carbapenem inactivation method and Blue-Carba test results). This heatmap was generated by using Morpheus online software using Euclidean distances (https://software.broadinstitute.org/morpheus/). Blue color indicates Positive or resistant; Red color indicates negative or sensitive, White color indicates Intermediate resistance. AK, Amikacin; AT, Aztreonam; CIP, Ciprofloxacin; LEV, Levofloxacin; IMI, Imipenem; MEM, Meropenem; AMC, Amoxicillin-clavulanic acid; CTR, Ceftriaxone; CX, Cefoxitin; ERT, Ertapenem; DOX, Doxycycline; TGC, Tigecycline

The 23, 17, 16, 5 XDR *A. baumannii*, *K. pneumoniae, P. aeruginosa* and *E. coli* isolates were clustered into 14, 10, 9 and 4 clusters, respectively (Figs. 2, 3, 4 and 5).

### ERIC-PCR analysis of some selected CR- GNB Isolates

Based on the heat map analysis, a total of 14, 10, 9 and 4 nonclonal clusters of *A. baumannii*, *K. pneumoniae*, *P. aeruginosa* and *E. coli*, 13, 8, 4 and 3 isolates were selected for genotypic relatedness analysis using ERIC-PCR. The ERIC-PCR was performed to the selected 28 CR- GNB isolates based on heatmap analysis to find out their genetic relatedness. Agarose gel electrophoresis of ERIC-PCR analysis of some selected carbapenem-resistant Gram-negative bacterial isolates is shown in Figure S1 (Supplementary data). The ERIC-PCR analysis confirmed non-clonal relatedness of most of the tested isolates (Supplementary data, Figures S2-S5). However, the dendrogram generated according to the calculated Jaccard similarity index showed genetic similarities of the following couples of: i) *A. baumannii,* isolates (AB4, AB13); (AB10, AB11); (AB2, AB6); (AB1, AB8), (AB7, AB9), (AB3,AB5) (Figure S2); ii) *P. aeruginosa* isolates (PA2, PA3) (Figure S3); iii) *E. coli* isolates (EC1, EC3) (Figure S4). For *K. pneumoniae* isolates results showed genetic identities of the five isolates namely, KP1, KP2, KP3, KP7 and KP8 (Figure S5). Although the genetically similar isolates were all collected from different patients, this suggests that these isolates shared a common source of infection. Aside from the respective isolates, all the other 28 tested CR-GNB isolates in this study were genetically dissimilar.

### MEM-PR combinations

The results of MEM in combination with PR is displayed in Table 3. Results showed that PR, in a concentration of 0.5 mg /ml, decreased MICs values of all of the tested XDR CR isolates (*n* = 28) and restored the susceptibility (MIC values were changed from resistance to sensitive values) of only 4 (14.3%) isolates (coded 84 s, 89 s, 50 s, 78 s). Interestingly, PR (1 mg/mL) when combined with MEM has completely (100%) restored the susceptibility of the tested isolates to MEM (Table 3).

### Effect of MEM + AK and MEP + TGC combinations

To reduce or eliminate CR in some CR-GNB isolates, the effects of different combinations of MEM with AK, and TGC were examined on 28 selected CR-GNB pathogens. The MEM + AK and MEM + TGC combinations revealed additive or indifferent effects against the tested CR-GNB isolates. The additive effects of MEM/AK and MEM/TGC were 92.8% and 71.4%, respectively, the indifferent effects of MEM/AK and MEM/TGC were 7.2% and 28.6%, respectively. While neither AK nor TGC showed synergism when combined with MEM, the calculated FIC index values are shown in Table 4.

**Table 4:** The calculated FIC index values of the MEM + AK and MEM + TGC combinations against 28 XDR CR pathogens

Isolate code	MIC of antimicrobial agents (µg/ml)/Susceptibility			MEM + AK		MEM + TGC	
	**MEM**	**AK**	**TGC**	**FIC index**	**Interpretation** ^a^	**FIC index**	**Interpretation** ^a^
AB-9G	256/R	1024/R	2/S	0.531	Additive	1.063	Indifferent
AB-109A	256/R	1024/R	0.5/S	0.531	Additive	1	Indifferent
AB-63 M	1024/R	64/R	0.5/S	0.516	Additive	1	Additive
AB-30G	512/R	16/S	2/S	1	Additive	0.75	Additive
AB-100 M	1024/R	512/R	2/S	0.75	Additive	0.75	Additive
AB-55 M	1024/R	512/R	1/S	0.625	Additive	1	Additive
AB-20G	256/R	64/R	1/S	1.5	Indifferent	0.75	Additive
AB-3 M	512/R	512/R	4/I	0.625	Additive	0.531	Additive
AB-14 M	128/R	256/R	0.5/S	1	Additive	1	Additive
AB-37 M	1024/R	128/R	2/S	0.516	Additive	0.75	Additive
AB-34 M	128/R	64/R	1/S	1	Additive	1	Additive
AB-13 M	512/R	64/R	2/S	0.531	Additive	1.031	Indifferent
AB-7G	512/R	64/R	2/S	0.531	Additive	0.75	Additive
KP-84S	64/R	256/R	4/I	0.625	Additive	0.75	Additive
KP-25S	1024/R	1024/R	4/I	0.75	Additive	0.531	Additive
KP-89S	128/R	256/R	4/I	1	Additive	0.75	Additive
KP-106S	512/R	512/R	2/S	0.625	Additive	1.031	Indifferent
KP-114S	1024/R	16/S	0.5/S	0.625	Additive	1	Additive
KP-92A	1024/R	64/R	4/I	0.516	Additive	0.531	Additive
KP-79A	512/R	64/R	4/I	0.531	Additive	0.531	Additive
KP-113S	512/R	512/R	1/S	0.625	Additive	1.063	Indifferent
PA-78S	128/R	64/R	256/R	1.5	Indifferent	1	Additive
PA-99S	1024/R	128/R	512/R	0.516	Additive	0.625	Additive
PA-50S	256/R	1024/R	1024/R	0.531	Additive	0.75	Additive
PA-54S	1024/R	512/R	128/R	0.625	Additive	0.516	Additive
EC-55A	512/R	16/S	1/S	1	Additive	1.031	Indifferent
EC-99A	256/R	16/S	0.5/S	0.625	Additive	1.25	Indifferent
EC-34R	64/R	1/S	0.5/S	0.625	Additive	1.063	Indifferent

*Abbreviations*: *FIC index* fractional inhibitory concentration index, *MEM* meropenem, *AK* amikacin, *TGC* tigecycline, *AB A. baumannii, KP K. pneumoniae, PA P. aeruginosa, EC E. coli*

^a^ FIC index was calculated using the lowest concentration of the respective antimicrobial agents at which the lowest value of FIC was achieved. Synergism ≤ 0.5, additive > 0.5—≤ 1, indifference > 1—≤ 4, antagonism > 4 [37]

## Discussion

The CR is considered as one of the most significant concerns to the public health worldwide [38, 39]. It has become a serious global threat, which limits the available therapeutic and treatment options. In this light, given that carbapenem is effective against almost all XDR GNB, it has gained clinical value as a last-resort treatment for serious bacterial infections. However, over the past few years, the prevalence of CR has steadily increased [40], and hence, our study aimed to find a new approach for combating the antibiotic resistance of XDR CR- GNB, the clinical relevant life-threatening pathogens. The clinical isolates were discharged from different clinical specimens of infected patients attending El Demerdash Tertiary Care Hospitals in Egypt. A total of 59 CR- GNB bacteria were recovered from 59 clinical specimens throughout the period of our study. where 21 (35.6%) were enterobacterial isolates and 38 (64.4%) were non- fermentative bacilli.

In our study, 23 (39%) *A. baumannii* isolates were recovered which may account for the pathogen's predominance within the recovered GNB isolates in our study. Furthermore, this data is of tremendous medical importance and a challenge from a medical standpoint as *A. baumannii* is one of the hard-to-treat organisms that cause nosocomial infections, this might be caused by its limitless ability to develop antimicrobial resistance due to the plasticity of its genome [41]. Our microbiological findings were consistent with some recent studies that found Enterobacteriaceae, especially, *K. pneumoniae* and *E. coli* together along with *A. baumannii*, and *P. aeruginosa* to be among the highest hazards within the GNB recovered from respiratory tract infections [42].

The antibiogram analysis demonstrated that all the 59 isolates [100%) had a high resistance level to various antimicrobials including carbapenems, this is beside the fact that the MIC of all isolates revealed that they are all CR and as a result, they are all classified as CR-XDR isolates [21], which was consistent with earlier studies that highlight the significant resistance of many CPs [18, 43]. CPs were initially screened for identifying MBLs utilizing a combined disk test. In our current study, 45.8% (27 out of 59) of the tested isolates were positive for class B carbapenemase. This showed that the carbapenem-resistance pattern may include other carbapenemase types, such as class A and class D oxacilinase (that were not inhibited by EDTA), This was almost near to a study carried out by Rakhi et al. that had reported 50% in the combined disk test for MBLs production [44].

To ensure the compliance with the most recent guidelines for carbapenemase production, the mCIM and blue- carba tests were additionally carried out on the 59 CP isolates. Our findings revealed that, mCIM test detected 52 (88.1%), while the blue- carba test detected 57 (96.6%) of CP isolates. This was comparable to the study conducted by Mabrouk et al. that had reported the lowest ratios 50% in the combined disk test for MBLs production followed by 92.3% for mCIM and the highest ratio of and 98.1% was recorded for blue-carba test [18]. Additionally, the blue- carba test results matched those of another study performed recently by Cordeiro-Moura et al. [45]. In the light of these reports, we can state that the blue-carba test demonstrated a high sensitivity and specificity for the detection of carbapenemase production and is thus regarded as a favorable tool for the quick detection of CPs in clinical settings [45]. We then tackled the determination of the prevalence of the carbapenemase genes, an essential character for the suppression of XDR-CR strains within clinical healthcare settings. After the phenotypic screening of XDR CR- GNB isolates, PCR was carried out utilizing the genomic DNA of each isolate and the specific primers for each CR gene. The choice of the five carbapenemase genes screened in this study was according to their prevalence among GNB and as they represent the three major classes of carbapenemases, counting, class A serine carbapenemases (*bla*KPC), class B metallo-β-lactamases (*bla*IMP, *bla*NDM and *bla*VIM) and class D serine carbapenemases (*bla*OXA-48) [1]. blaKPC was the most predominant CR gene in our findings (67.8%) that was equivalent to the results of a recent study conducted by Li and his colleagues [46], next was blaOXA-48 (50.8%), then *bla*VIM that was noticed in (27.1%) of the isolates. However, neither *bla*IMP, *bla*VIM or *bla*NDM was found in any isolate which was in accordance with the results of a study performed by Mabrouk et al. who reported high frequency of *bla*KPC (63.5%) [18]. On the contrary, our results differed from those from a study performed in Zagazig hospital in Egypt, which indicated the clonal spread of *bla*OXA-23 to be (90%), next was *bla*NDM (66.7%) and last was *bla*GES (50%) in CR *A. baumannii* [47]. The discrepancy between the relevant study and ours might be related to other variables including geographical and patient factors. Strikingly, the presence of two carbapenemase genes was observed in 19 (32.2%) of the 59 carbapenemase-positive isolates, and their distribution was as follows: 16 isolates co- harbored *bla*KPC and *bla*OXA-48, 2 isolates co-harbored *bla*KPC and *bla*VIM, and 2 isolates co-harbored *bla*OXA-48 and *bla*VIM. Furthermore the 3 carbapenemase genes that were *bla*KPC, *bla*OXA-48 and *bla*VIM were co-harbored in 4 (6.78%) isolates, this was in an accordance to a recent study by Mabrouk et al. [18].

A dendrogram that showed heatmap signature of the CR-GNB isolates was generated in an effort to shed more light on the phenotypic relatedness of the isolates based on their antimicrobial resistance patterns and their capacity to produce carbapenemase enzymes. Isolates showing similar heatmap signatures were mostly *A. baumannii* followed by *P. aeruginosa*, indicating that they might be nosocomially transmitted as has been previously reported [43, 48]. The ability of ERIC-PCR as a genotyping tool to investigate epidemics of hospital-acquired infections rely on its capability to epidemiologically relate the isolates obtained during a nosocomial outbreak and to determine whether the involved isolates are genetically related or descended from different strains [49]. Both phenotypic and genotypic relatedness have been performed in our study by using heatmap and ERIC PCR analysis, respectively to evaluate clonal diversity and to select the diverse isolates for the next experiments. Using strain typing in infectious disease control decisions in our hospitals is based on several hypotheses, including whether the isolates linked to the outbreak are descendants of a single clone, whether such isolates will have the same genotype, and whether the isolates from unrelated epidemiological cases will have different genotypes [50]. The dendrogram obtained from the genomic DNA products of the ERIC-PCR revealed that the majority of the tested isolates were not clonal. However, some isolates showing genetic relatedness giving the possibility of their common etiology of our clinical setting. It is abundantly clear that one cannot rely solely on the results of phenotypic data for epidemiologic studies and genotypic analysis must be carried out in order to obtain more precise results. This finding might point to clonal expansion and microbial colonization from various sources [48] and therefore, extensive prevention and decontamination control measures should be undertaken in the respective clinical setting to avoid clonal expansion of these clinically-relevant pathogens.

Addituonally, the achieved results highlighted the critical demand for a new drug combination scenario in addition to speeding up the development of new infection control approaches against XDR- CR clinically relevant GNB pathogens. In the current study, a new scenario has been performed for reaching new alternative treatment options for the control of infections caused by XDR CR- GNB isolates, the most life-threatening pathogens. This was achieved by evaluating the use of MEM, the most commonly used antimicrobial agent for the treatment of CR-GNB infections together with either TGC or AK or non- antibiotics such as PR. Several studies highlighted the fact that the combination of a carbapenem with either TGC or aminoglycoside or colistin seems to have a privilege over monotherapy with either TGC or colistin or aminoglycosides alone for combating CR- GNB pathogens [51–53]. The justification for combining two or more antibiotics against CR- GNB is to increase bacterial eradication rates while lowering the emergence of resistance [54]. In our study, combination of MEM with either AK or TGC was tested on 28 XDR CR- GNB showed either additive or indifference effects, while none of the tested combinations showed synergistic effect against the tested clinically relevant isolates. However, our results were different from previously conducted studies that recorded a high synergistic effect with either combination of MEM/AK or MEM/TGC against CR- GNB pathogens [55–59]. Our findings were similar to another study conducted by Antonelli et al. which revealed the synergistic/additive effects among only 16.1% of the tested strains [60]. Also, our findings were in an accordance with a previous study that has revealed the scarcity of synergism when using MEM-TGC combination in carbapenemase-producing Enterobacteriaceae (CPE) strains by time-kill curve analysis [61]. Another recent study revealed the antagonism between TGC and MEM tested against KPC-producing *K. pneumoniae* infections [62]. Based on our findings along with recently published records, MEM combination together with AK or TGC should be used with caution due to their lack of synergism.

On the other hand, several successful combinations of MEM with non-antibiotics against CR-GNB were previously reported [63–65]. However, there have been few investigations on using non-selective beta-blockers, including PR, in combination with antimicrobial agents [7] or carvedilol alone [66]. In our current study, combination of MEM with PR showed promising results and successfully overcame bacterial resistance of XDR CR clinical isolates. MEM combination with PR at concentration 0.5 mg/mL significantly increase the susceptibility of the tested XDR CR- GNB pathogens isolates to MEM (MDF ranged from 8–64]. While the MIC of only four isolates 14.3% was declined from 64 and 128 µg/ml (e.g., resistant phenotype) to 2 µg/ml (e.g., sensitive phenotype), respectively.

However, MEM combination with PR at concentration 1 mg/mL significantly increase the susceptibility of the tested XDR CR- GNB isolates by 100% (MDF ranged from 64–1024), while 85.7% of the tested isolates was changed from resistant phenotype to sensitive phenotype e.g., PR restored their susceptibility. These results could be attributed to the efflux pump inhibitory action of PR or its antibacterial action that has been previously investigated [67, 68]. Our results were in agreement with another study previously reported, where it has been found that combination of CIP or LEV with PR effectively conquered bacterial resistance of XDR- and PDR-*A. baumannii* clinical isolates [12].

## Conclusion

This research highlighted the high prevalence of XDR CR- GNB clinically relevant pathogens. MEM combination together with AK or TGC should be used with caution due to their lack of synergism. Our results revealed that PR at a concentration of 1 mg/mL restored susceptibility of all selected XDR-CR clinical isolates. Moreover, our findings support the use of high-quality in vitro research to investigate potentially effective combination regimens for use in clinical practice and to guide the choice of antibiotic treatments to strengthen the armamentarium against CR- GNB. In addition, further pharmacokinetic/pharmacodynamics studies are required to direct the utilization of these new promising combinations in the face of these deadly clinical pathogens.

## Supplementary Information

**Additional file 1:** **Figure S1.** Agarose gel electrophoresis of ERIC-PCR analysis of some selected carbapenem-resistant Gram-negative bacterial isolates; lanes A1-A13 were ERIC PCR analysis of 13 *Acinetobacter baumannii* clinical isolates (coded AB1-AB13); lanes P1-P4 were ERIC PCR analysis of 4 *Pseudomonas aeruginosa* clinical isolates (coded PA1-PA-4); lanes K1-K8 were ERIC PCR analysis of 8 *Klebsiella pneumoniae* clinical isolates (coded KP1-KP8); lanes E1-E3 were ERIC PCR analysis of 3 *E. coli* clinical isolates (coded EC1-EC3). lane L, a gene Ruler 1 kb ladder (Thermo Scientific™ Oxoid™, Loughborough, UK). **Figure S2.** Dendrogram generated from ERIC-PCR genomic DNA products of 13 carbapenem-resistant *A. baumnnii* bacterial isolates (AB1-AB13). **Figure S3.** Dendrogram generated from ERIC-PCR genomic DNA products of 8 carbapenem-resistant *K. pneumoniae* bacterial isolates (KP1-KP8). **Figure S4.** Dendrogram generated from ERIC-PCR genomic DNA products of 8 carbapenem-resistant *P. aeruginosa* bacterial isolates (PA1-PA4). **Figure S5.** Dendrogram generated from ERIC-PCR genomic DNA products of 8 carbapenem-resistant *E. coli* bacterial isolates (EC1-EC3).

## Data Availability

All data generated or analyzed during this study are included in this published article in the main manuscript and supplementary file.

## Abbreviations

AK: Amikacin
*Bla*KPC: The gene coded for *Klebsiella pneumoniae* carbapenemases (KPC)
*bla*NDM: The gene coded for New Delhi metallo-β-lactamase (NDM)
*Bla*IMP: The gene coded for the imipenem-resistant *Pseudomonas*-type carbapenemases (IMP)
*bla*VIM: A gene coded for Verona integron-encoded metallo-β-lactamase (VIM)
*bla*OXA − 48: The gene coded oxacillinase (OXA-48-like) types
CLSI: Clinical and laboratory standard institute
CDT: Combined disk test
CPs: Carbapenemase producers
CR- GNB: Carbapenem resistant Gram-negative bacteria
EDTA: Ethylene diamine tetra acetic acid
ERIC-PCR: Enterobacterial repetitive intergenic consensus-PCR
ESBLs: Extended-spectrum beta-lactamases
MEM: Meropenem
MBLs: Metallo-beta lactamases
mCIM: Modified carbapenem inactivation method
MDF: MIC decrease factor
MIC: Minimum inhibitory concentration
ND: Not determined
PCR: Polymerase chain reaction
PR: Propranolol
TGC: Tigecycline
XDR: Extensive drug resistant
