# Supplementary material for: Propranolol restores susceptibility of XDR Gram-negative pathogens to meropenem and Meropenem combination has been evaluated with either tigecycline or amikacin
Source: BMC Microbiol. 2023 Jul 22;23:195. doi: 10.1186/s12866-023-02934-6 (PMC10362616; doi:10.1186/s12866-023-02934-6)
Supplement: Supplementary file 1 — Additional file 1: Figure S1. Agarose gel electrophoresis of ERIC-PCR analysis of some selected carbapenem-resistant Gram-negative bacterial isolates; lanes A1-A13 were ERIC PCR analysis of 13 Acinetobacter baumannii clinical isolates (coded AB1-AB13); lanes P1-P4 were ERIC PCR analysis of 4 Pseudomonas aeruginosa clinical isolates (coded PA1-PA-4); lanes K1-K8 were ERIC PCR analysis of 8 Klebsiella pneumoniae clinical isolates (coded KP1-KP8); lanes E1-E3 were ERIC PCR analysis of 3 E. coli clinical isolates (coded EC1-EC3). lane L, a gene Ruler 1 kb ladder (Thermo Scientific™ Oxoid™, Loughborough, UK). Figure S2. Dendrogram generated from ERIC-PCR genomic DNA products of 13 carbapenem-resistant A. baumnnii bacterial isolates (AB1-AB13). Figure S3. Dendrogram generated from ERIC-PCR genomic DNA products of 8 carbapenem-resistant K. pneumoniae bacterial isolates (KP1-KP8). Figure S4. Dendrogram generated from ERIC-PCR genomic DNA products of 8 carbapenem-resistant P. aeruginosa bacterial isolates (PA1-PA4). Figure S5. Dendrogram generated from ERIC-PCR genomic DNA products of 8 carbapenem-resistant E. coli bacterial isolates (EC1-EC3). [file 12866_2023_2934_MOESM1_ESM.docx]

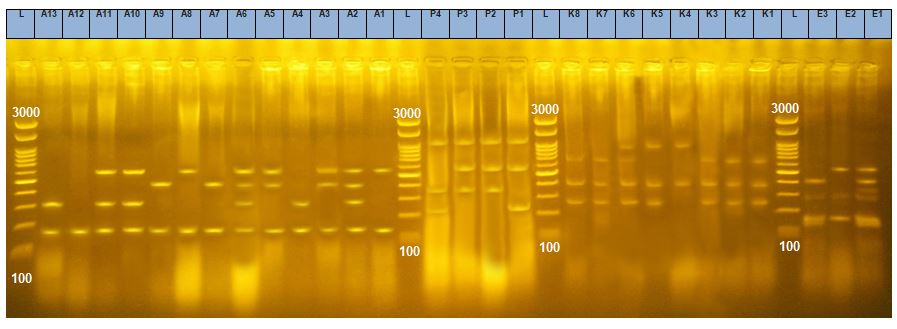


**Figure S1.** Agarose gel electrophoresis of ERIC-PCR analysis of some selected carbapenem-resistant Gram-negative bacterial isolates; lanes A1-A13 were ERIC PCR analysis of 13 *Acinetobacter baumannii* clinical isolates (coded AB1-AB13); lanes P1-P4 were ERIC PCR analysis of 4 *Pseudomonas aeruginosa* clinical isolates (coded PA1-PA-4); lanes K1-K8 were ERIC PCR analysis of 8 *Klebsiella pneumoniae* clinical isolates (coded KP1-KP8); lanes E1-E3 were ERIC PCR analysis of 3 *E. coli* clinical isolates (coded EC1-EC3). lane L, a gene Ruler 1 kb ladder (Thermo Scientific™ Oxoid™, Loughborough, UK);


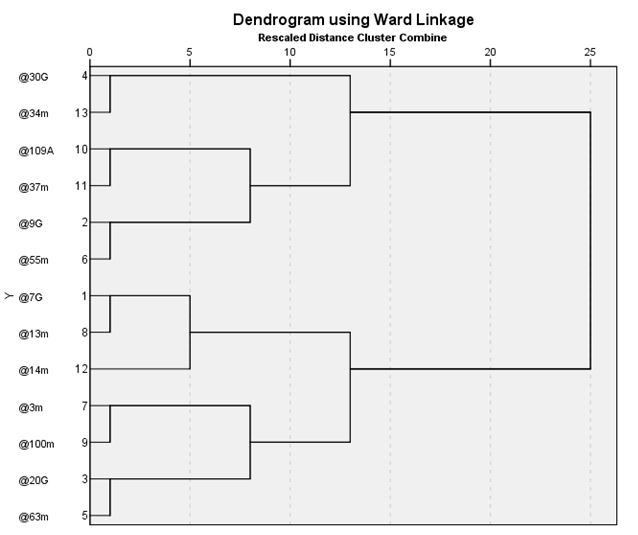


**Figure S2.** Dendrogram generated from ERIC-PCR genomic DNA products of 13 carbapenem-resistant *A. baumnnii* bacterial isolates (AB1-AB13).


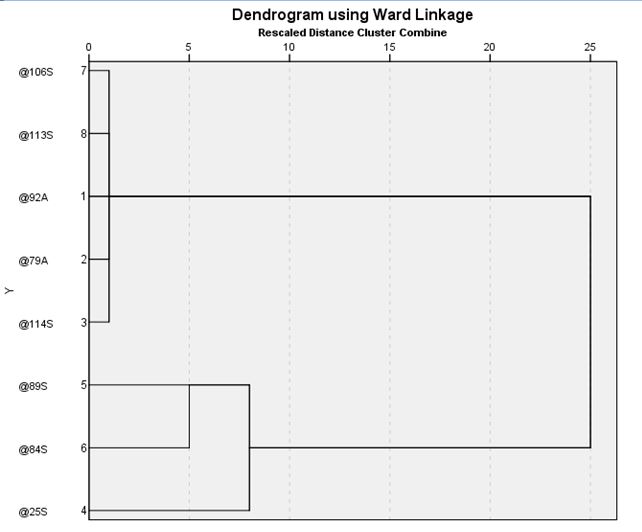


**Figure S3.** Dendrogram generated from ERIC-PCR genomic DNA products of 8 carbapenem-resistant *K. pneumoniae* bacterial isolates (KP1-KP8)


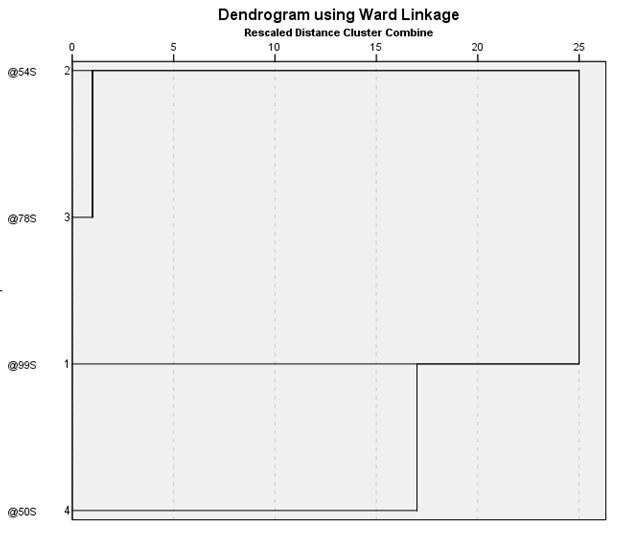


**Figure S4.** Dendrogram generated from ERIC-PCR genomic DNA products of 8 carbapenem-resistant *P. aeruginosa* bacterial isolates (PA1-PA4)


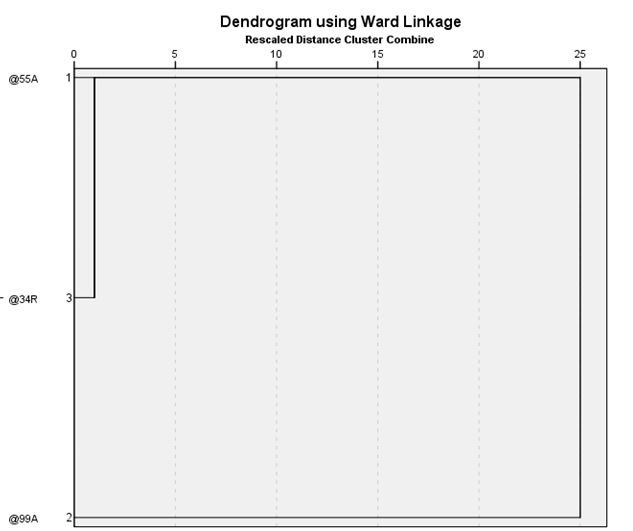


**Figure S5.** Dendrogram generated from ERIC-PCR genomic DNA products of 8 carbapenem-resistant *E. coli* bacterial isolates (EC1-EC3)
